# Supplementary material for: FXR and BET signaling orchestrate to protect β cells
Source: bioRxiv. 2026 Apr 14:2026.04.10.716420. Preprint. [Version 1] doi: 10.64898/2026.04.10.716420 (PMC13105080; doi:10.64898/2026.04.10.716420)
Supplement: Supplement 1 [file NIHPP2026.04.10.716420v1-supplement-1.pdf]

bioRxiv preprint doi: https://doi.org/10.64898/2026.04.10.716420; this version posted April 14, 2026. The copyright holder for this preprint (which was not certified by peer review) is the author/funder, who has granted bioRxiv a license to display the preprint in perpetuity. It is made available under aCC-BY 4.0 International license.

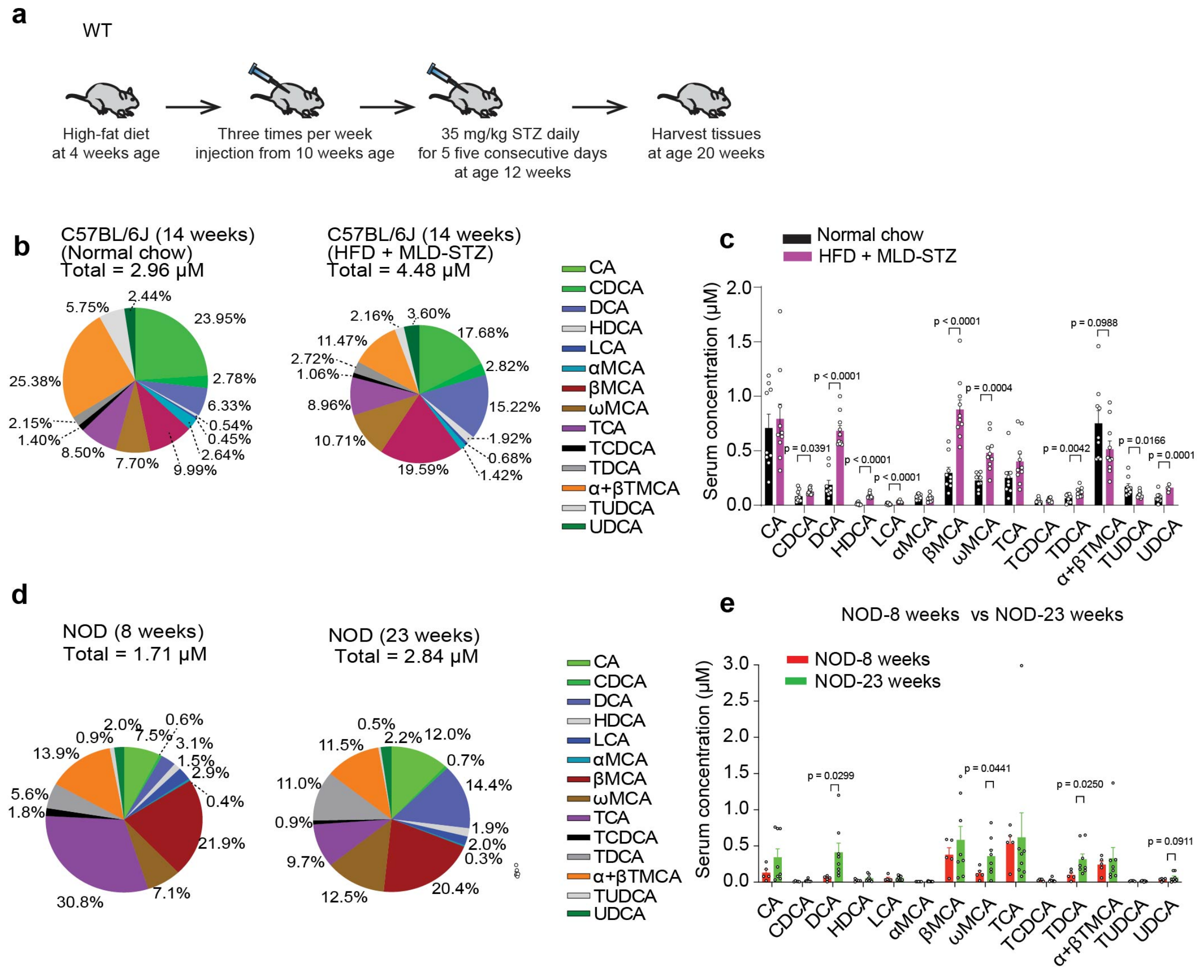

Supplementary Fig.1 Dysregulation of bile acid signaling in HFD + MLD-STZ and NOD mouse models.
a, Schematic of the T2D model. C57BL/6J mice were fed a high-fat diet (HFD) starting at 4 weeks of age. Pre-treatment was initiated at 10 weeks, followed by multiple low-dose STZ (MLD-STZ) administration for five consecutive days starting at 12 weeks. **b**, Comparison of serum bile acid (BA) profiles shown as total levels and relative composition between non-diabetic, age-matched male C57BL/6J mice fed a normal chow diet (left pie chart) and diabetic, age-matched male C57BL/6J mice subjected to HFD + MLD-STZ treatment (right pie chart). Serum samples were collected 14 days after the final STZ injection. n = 9 (normal chow), n = 10 (HFD + MLD-STZ).**c**, Comparison of serum individual BA amount in non-diabetic, normal chow-fed C57BL/6J versus diabetic C57BL/6J on HFD + MLD-STZ. n = 9 for C57BL/6J on normal chow, n = 10 for C57BL/6J on HFD + MLD-STZ. **d**, Serum bile acid (BA) profile of NOD mice (8 weeks or 23 weeks, female) measured by HPLC-MS/MS. e, Comparison of serum BA amounts in female NOD mice at 8 weeks vs 23 weeks. 8 weeks NOD n = 5, 23 weeks NOD n = 8. Statistical analysis was performed using unpaired two-tailed Student's t-test. Error bars represent mean ± SEM.

bioRxiv preprint doi: https://doi.org/10.64898/2026.04.10.716420; this version posted April 14, 2026. The copyright holder for this preprint (which was not certified by peer review) is the author/funder, who has granted bioRxiv a license to display the preprint in perpetuity. It is made available under aCC-BY 4.0 International license.

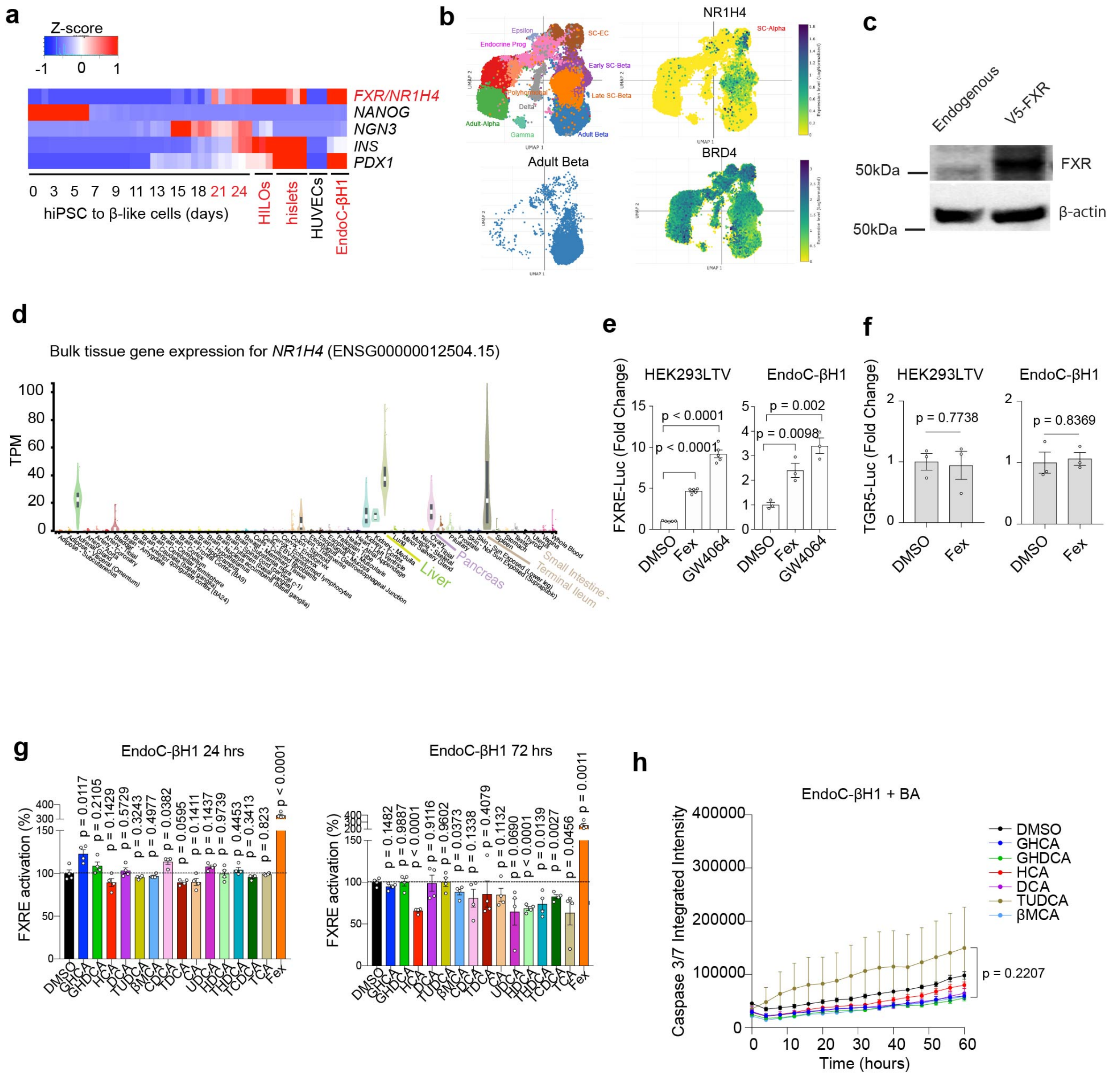

Supplementary Fig. 2. FXR signaling in human β cells.

a, Heatmap of FXR gene expression and β cell marker genes in human iPSCs during their stepwise differentiation into HILOs compared to human islets, HUVEC cells, and EndoC-βH1. **b**, *NR1H4* gene expression in human islets and hPSC-derived pancreatic cells. Data was obtained from single cell Portal Broad institute (Functional, metabolic and transcriptional maturation of human pancreatic islets derived from stem cells). **c**, Representative image of FXR Western blot for endogenous and DOX-induced FXR overexpression system in EndoC-βH1 human β cell line. **d**, *NR1H4* expression in human tissues. Data was obtained from GTEx Portal. **e**, FXR reporter assay after 24 hours of treatment with DMSO, Fex (10 μM) or GW4064 (10 μM) in HEK293LTV and EndoC-βH1 cells. n = 3/each. **f**, TGR5-luciferase in EndoC-βH1 and HEK293LTV cell lines containing the TGR5 luciferase reporter after 24 hours treatment with the FXR-specific agonist Fex. n = 3/each. **g**, FXRE (FXR responsive element)-luciferase reporter signal of different BAs (10 μM each for indicated time) and Fex on EndoC-βH1 cells after 24 hours and 72 hours treatment. n = 4/each. **h**, Real-time imaging of caspase 3/7 green intensity signal in EndoC-βH1 cell line for 60 hours incubation with BAs (10 μM each for indicated time) without inflammatory cytokine IL-1β. n = 4/each. Statistical analyses were performed using unpaired Student's two-tailed t-tests for (f), one-way ANOVA with Tukey's multiple-comparison test for (e, g), and two-way ANOVA for (h). Error bars represent mean ± SEM.

bioRxiv preprint doi: https://doi.org/10.64898/2026.04.10.716420; this version posted April 14, 2026. The copyright holder for this preprint (which was not certified by peer review) is the author/funder, who has granted bioRxiv a license to display the preprint in perpetuity. It is made available under aCC-BY 4.0 International license.

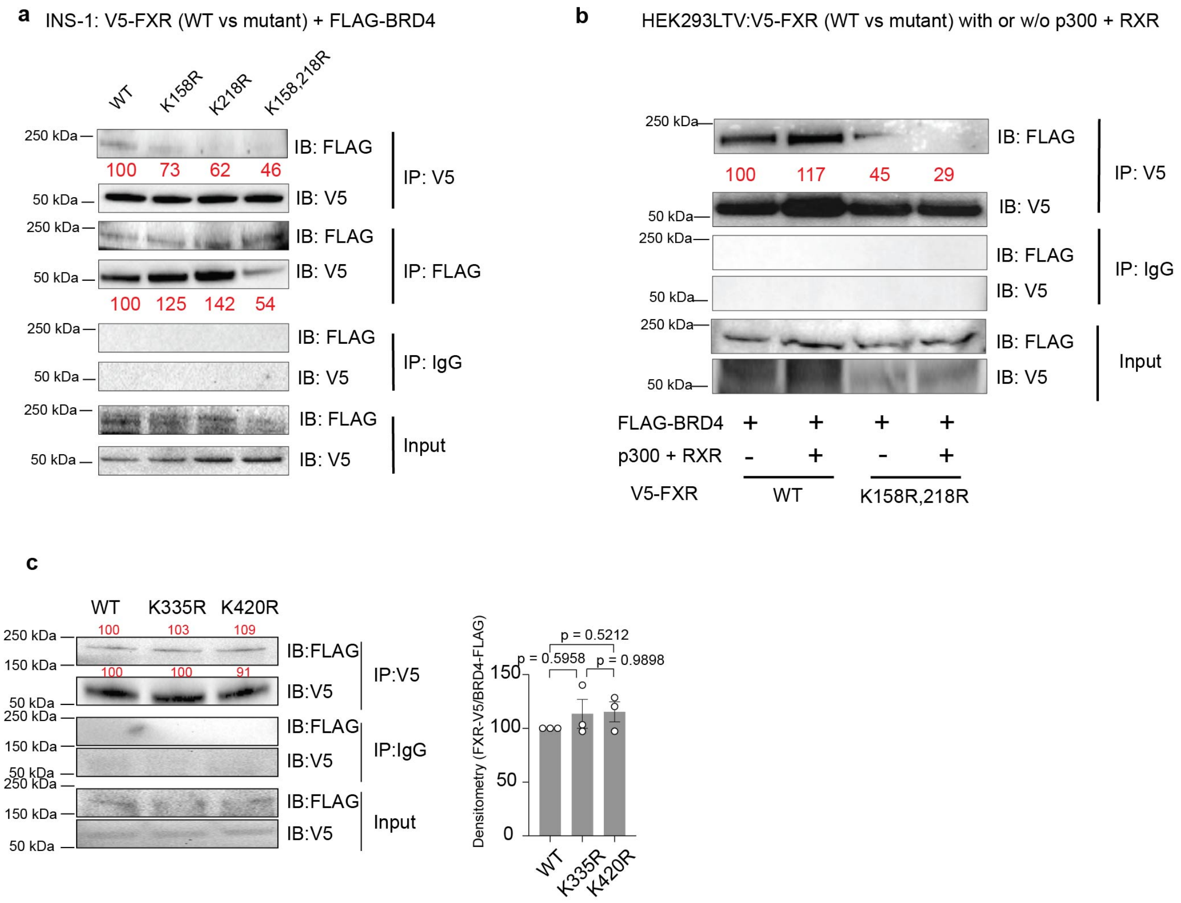

Supplementary Fig. 3. Physical interaction between FXR and BRD2, BRD3, or BRD4.

a, IB: FLAG-BRD4 or V5 in V5-FXR (control), V5-FXR (K158R), V5-FXR (K218R), V5-FXR (K158R/K218R) pull-down (IP:V5, FLAG or IgG) protein complexes from INS-1 cells. **b**, Co-IP of FLAG-BRD4 or V5-FXR + p300 + RXR in HEK293LTV cells after co-transfected with FLAG-BRD4 and V5-FXR (WT) or V5-FXR (mut, K158R/K218R). **c**, Immunoblot (IB) of FLAG or V5 in V5-FXR (WT), V5-FXR (K335R), V5-FXR (K420R) pull-down (IP:V5) protein complexes from EndoC-βH1 cells. Red numbers above the blots indicate densitometry values presented as percent relative to FXR-WT which is set as 100. The bar graph represents densitometric analyses of FXR-V5/BRD4-FLAG ratios from three independent experiments. Statistical analysis was performed using one-way ANOVA with Tukey's multiple-comparison test. Error bars represent mean ± SEM

bioRxiv preprint doi: https://doi.org/10.64898/2026.04.10.716420; this version posted April 14, 2026. The copyright holder for this preprint (which was not certified by peer review) is the author/funder, who has granted bioRxiv a license to display the preprint in perpetuity. It is made available under aCC-BY 4.0 International license.

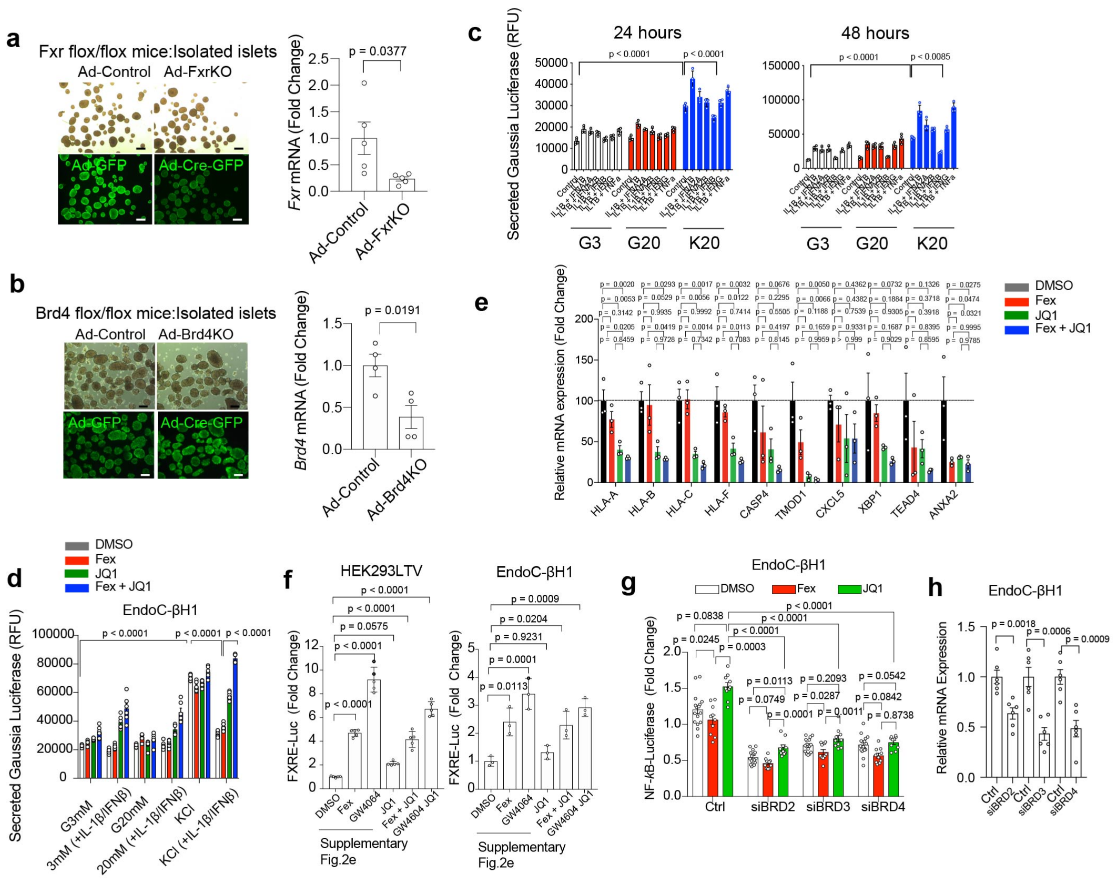

Supplementary Fig. 4. FXR agonist and BET inhibitor synergistically regulate β cell insulin secretion via FXR and BRD4 under inflammatory stress.

a, Upper panel: Optical and fluorescence image of isolated islets from Fxr flox/flox mice with adenovirus-mediated GFP (Ad-Control) or Cre-GFP (Ad-FxrKO) expression. Bottom panel: qPCR analyses of *Fxr* gene expression in Ad-Control and Ad-FxrKO islets. Scale bar = 100 µm. **b**, Upper panel: Optical and fluorescence image of isolated islets from Brd4 flox/flox mice with adenovirus-mediated GFP (Ad-Control) or Cre-GFP (Ad-Brd4KO) expression. Lower panel: qPCR analyses of *Fxr* gene expression in Ad-Control and Ad-Brd4KO islets. Scale bar = 100 µm. **c**, Gaussia-luciferase reporter assay for insulin secretion of EndoC-βH1 cells after 48 hours incubation with DMSO, Fex (10 µM), JQ1 (500 nM) or Fex (10 µM) + JQ1 (500 nM) and with or without 24 hours or 48 hours treatment with the inflammatory cytokines IL-1β (10 ng/ml) and IFNα2A (10 ng/ml), IFNα2B (10 ng/ml), IFNβ (10 ng/ml), IFNγ (10 ng/ml) or TNFα (10 ng/ml). n = 6/each. **d**, qPCR analyses of indicated genes in EndoC-βH1 cells pretreated with DMSO, Fex (10 µM), and/or JQ1 (500 nM) for 72 hours and with IL-1β (10 ng/ml) + IFNγ (10 ng/ml) for 48 hours. n = 3/each. **e**, Gaussia-luciferase reporter assay for insulin secretion corresponding to Fig. 3e. **f**, FXR reporter assay of EndoC-βH1 and HEK293LTV cells after 24 hours of treatment with Fex (10 µM), GW4604 (10 µM) and/or JQ1 (500 nM). n=3/each. **g**, NF-*k*B luciferase activity in EndoC-βH1 cells transfected siRNA against *BRD2* (siBRD2), *BRD3* (siBRD3), or *BRD4* (siBRD4) respectively 96 hours prior of the Luciferase assay. Fex (10 µM) or JQ1 (500 nM) were treated for 48 hours and IL-1β (10 ng/ml) for 24 hours prior of the Luciferase assay. **h**, qPCR analyses of BRD2, BRD3, or BRD4 48 hours after siRNA transfection in EndoC-βH1 cells. Statistical analyses were performed using unpaired two-tailed Student's t-tests for (a, b, h) and one-way ANOVA with Tukey's multiple-comparison test for (c–g). Error bars represent mean ± SEM.

bioRxiv preprint doi: https://doi.org/10.64898/2026.04.10.716420; this version posted April 14, 2026. The copyright holder for this preprint (which was not certified by peer review) is the author/funder, who has granted bioRxiv a license to display the preprint in perpetuity. It is made available under aCC-BY 4.0 International license.

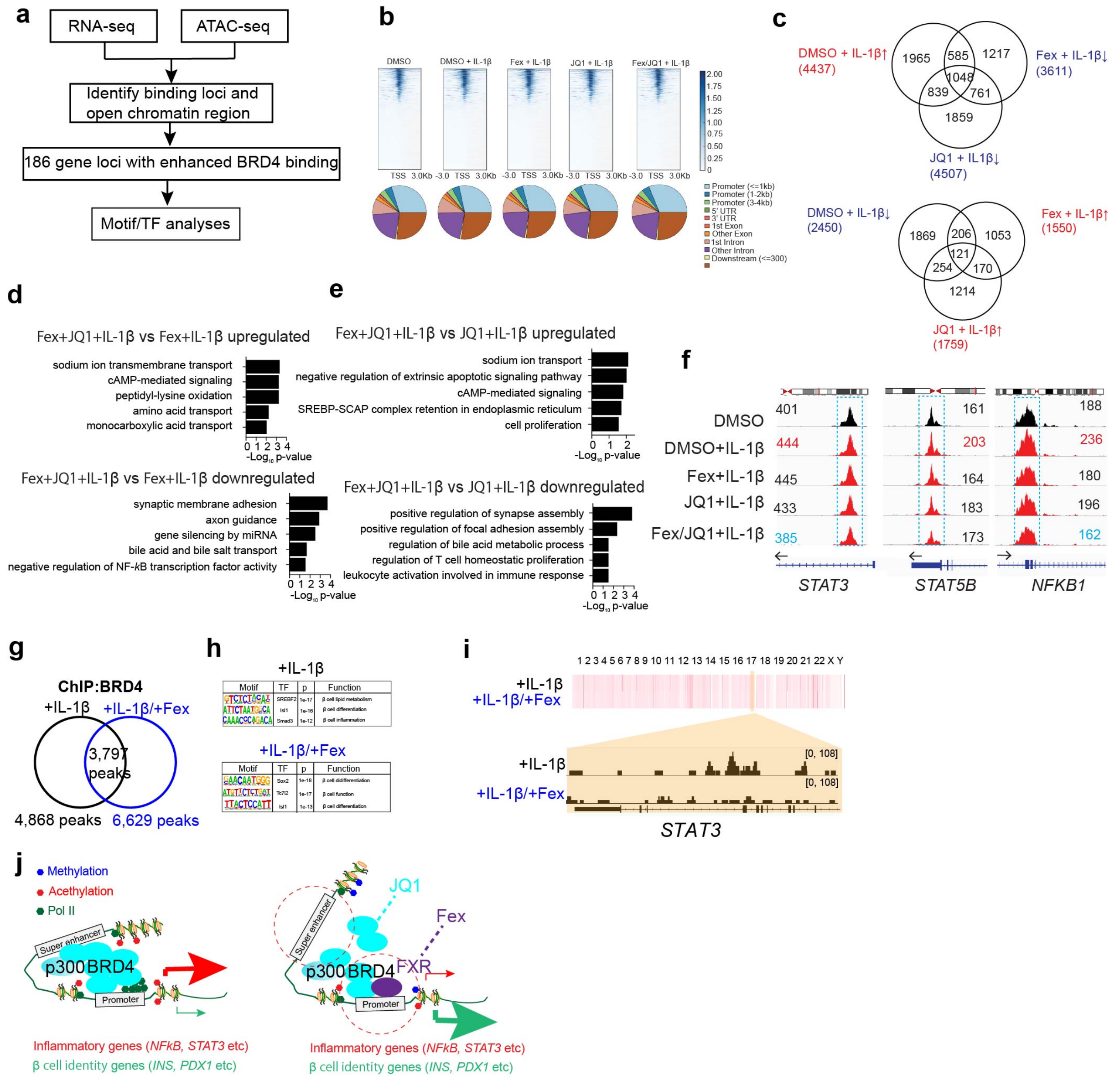

Supplementary Fig. 5. ATAC-seq and BRD4 ChIP-seq analyses in human β cells.

a, Schematic of RNA-seq, ATAC-seq and ChIP-seq of BRD4 binding loci identification in EndoC-βH1 cells. **b**, Chromatin accessibility at sites in response to DMSO, DMSO + IL-1β, Fex + IL-1β, JQ1 + IL-1β, or Fex + JQ1 + IL-1β in EndoC-βH1 cells. **c**, Number of peaks significantly up or downregulated by IL-1β, while preserved by Fex or JQ1. **d, e**, GO of synergistically up or down regulated by Fex + JQ1 compared to single treatment of Fex (**d**) or JQ1 (**e**) under the stress of IL-1β. **f**, Browser tracks showing ATAC-seq data with indicated treatments at indicated loci. **g**, Venn diagram for BRD4 binding regions at IL-1β (10 ng/ml, 24 hours) with or without Fex (10 μM, 48 hours) treatment in EndoC-βH1 cells. **h**, Motif analysis of BRD4 binding regions in EndoC-βH1 cells. **i**, Genome browser view of BRD4 binding to the *STAT3* locus in IL-1β (10 ng/ml, 24 hours) with or without Fex (10 μM, 48 hours) treated EndoC-βH1 cells. **j**, Model of BRD4 and FXR mediated transcriptional regulation for inflammatory and β cell identity genes.

bioRxiv preprint doi: https://doi.org/10.64898/2026.04.10.716420; this version posted April 14, 2026. The copyright holder for this preprint (which was not certified by peer review) is the author/funder, who has granted bioRxiv a license to display the preprint in perpetuity. It is made available under aCC-BY 4.0 International license.

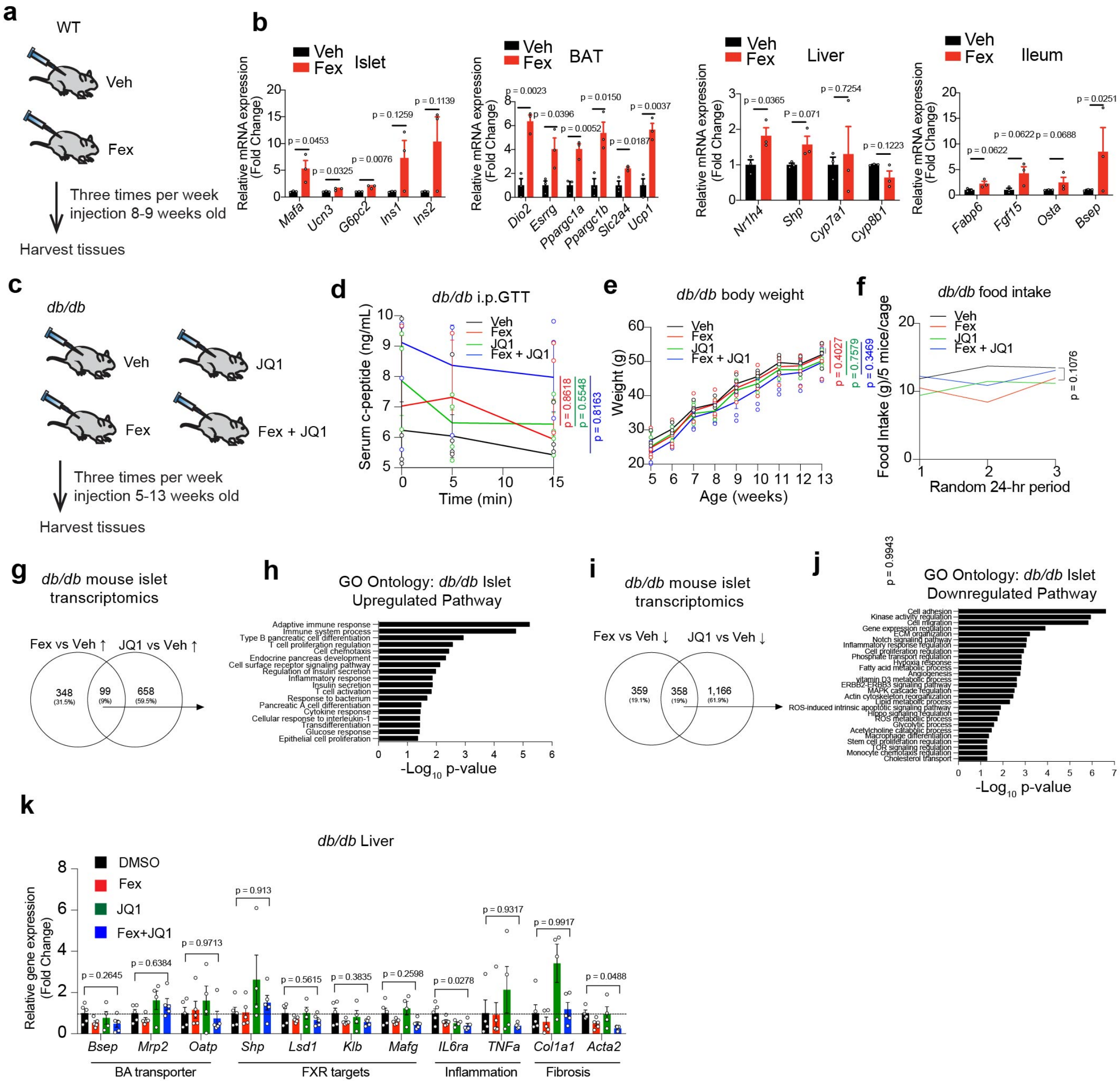

Supplementary Fig. 6. FXR agonist and BET inhibitor do not alter body weight gain in *db/db* mice.
a, Scheme of mouse experiments. Veh or Fex (50 mg/kg) are i.p. injected three times a week between 8 to 9 weeks old C57BL6J male mice and tissue are harvested 24 hours after the last i.p.. **b,** qPCR analyses of indicated genes in isolated islets, brown adipose tissue (BAT), liver and ileum. n = 5 each. **c,** Scheme of mouse experiments. Veh, Fex (50 mg/kg), JQ1 (10 mg/kg), or Fex (50 mg/kg) + JQ1 (10 mg/kg) are i.p. injected daily from 5 weeks to 13 weeks old *db/db* male mice and tissue are harvested. **d,** Glucose tolerance test for serum c-peptide of *db/db* after 8-week i.p. treatment with Veh, Fex, JQ1 or Fex+JQ1. n = 5/each. **e**, Body weight of *db/db* mice during the 8-week i.p. treatment with Veh, Fex, JQ1 or Fex+JQ1. n=5/each. **f**, Food consumption of *db/db* mice at three random 24 hour periods during the 8-week i.p. treatment with Veh, Fex, JQ1, or Fex+JQ1. n = 5/each. **g**, Venn diagram of differentially upregulated genes in pancreatic islets isolated from *db/db* mice treated with Fex or JQ1. n=3/each. **h**, Gene ontology pathway analysis of the common 99 differentially upregulated genes in pancreatic islets isolated from *db/db* mice treated with Fex or JQ1. n = 3/each. **i**, Venn diagram of differentially downregulated genes in pancreatic islets isolated from *db/db* mice treated with Fex or JQ1. n = 3/each. **j**, Gene ontology pathway analysis of the common 358 differentially downregulated genes in pancreatic islets isolated from *db/db* mice treated with Fex or JQ1. n = 3/each. **k**, qPCR analyses of indicated genes in liver of *db/db* at 13 weeks of age. Statistical analyses were performed using unpaired two-tailed Student's t-tests for (b) one-way ANOVA with Tukey's multiple-comparison test for (f, k) and two-way ANOVA for (d, e). Error bars represent mean ± SEM.

bioRxiv preprint doi: https://doi.org/10.64898/2026.04.10.716420; this version posted April 14, 2026. The copyright holder for this preprint (which was not certified by peer review) is the author/funder, who has granted bioRxiv a license to display the preprint in perpetuity. It is made available under aCC-BY 4.0 International license.

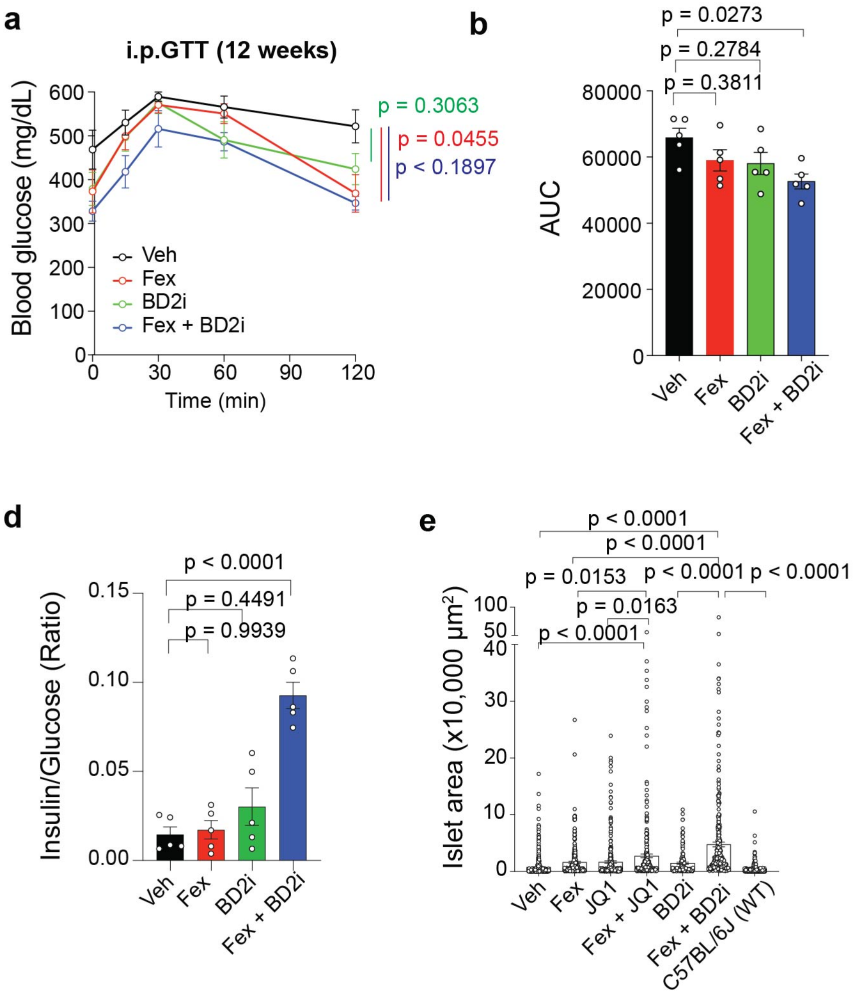

Supplementary Fig. 7. FXR agonist and iBET-BD2 inhibitor improve glucose homeostasis in *db/db* mice.
a, Intraperitoneal glucose tolerance test on the 12-week old of i.p. treatment with Veh, Fex, JQ1 or Fex + JQ1 combination. n = 5/each. **b**, Area under the curve (AUC) for the intraperitoneal glucose tolerance test at the 8-week of i.p. treatment with Veh, Fex, JQ1 or Fex + JQ1 combination. n = 5/each. **c**, Serum insulin/glucose index of 12 week old *db/db* with i.p. treatment with Veh, Fex, JQ1 or Fex + JQ1 combination. n = 5/each. **d**, Serum insulin/glucose index of *db/db* mice i.p. treatment of Veh, Fex, JQ1 or Fex + JQ1 combination. n = 5/each. **e**, Area of islets from H&E staining of *db/db* mouse pancreas after 8-weeks of i.p. treatment with vehicle, Fex, JQ1, BD2i, Fex + JQ1 or Fex + BD2i combination. Two histology slides per condition. Statistical analyses were performed using two-way ANOVA for (a) and one-way ANOVA with Tukey's multiple-comparison test for (b–e). Error bars represent ± SEM.

bioRxiv preprint doi: https://doi.org/10.64898/2026.04.10.716420; this version posted April 14, 2026. The copyright holder for this preprint (which was not certified by peer review) is the author/funder, who has granted bioRxiv a license to display the preprint in perpetuity. It is made available under aCC-BY 4.0 International license.

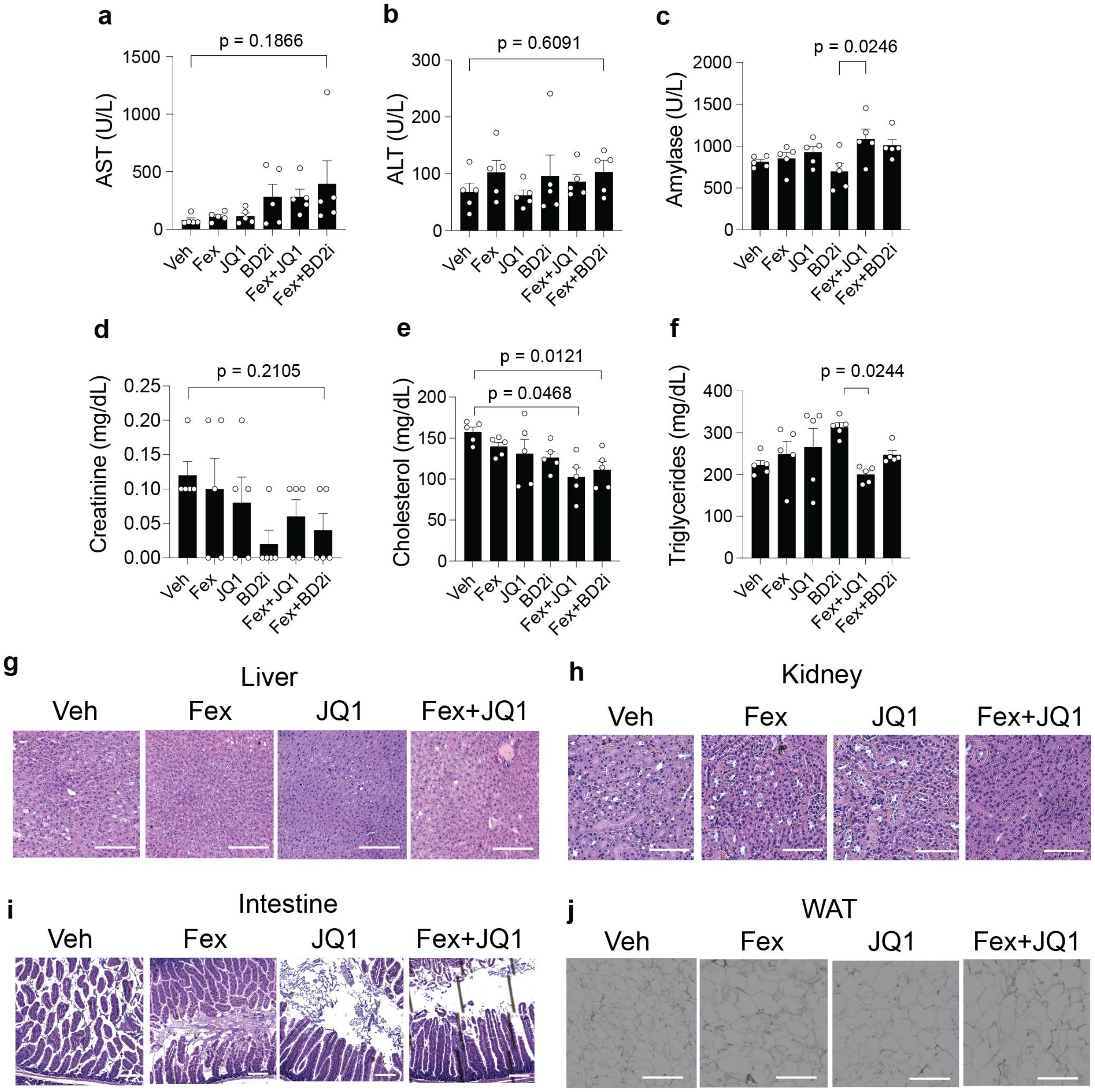

Supplementary Fig. 8. Impact of serum parameters by FXR agonist and BET inhibitors in *db/db* mice
(**a-f**), Serum metabolic parameters are measured at 13 weeks old *db/db* mice after treating with indicated drugs with daily i.p. injection from 5 weeks old to 13 weeks old. AST (U/L) (**a**), ALT (U/L) (**b**), Amylase (U/L) (**c**), Creatinine (mg/dL) (**d**), Cholesterol (mg/dL) (**e**) and Triglycerides (**f**). (**g-j**) Representative images for H&E stain for the liver (g), kidney (h), intestine (i) and white adipose tissue (WAT) (j) of *db/db* mice after the 8-week of i.p. treatment with Veh, Fex, JQ1 or Fex + JQ1 combination. scale bar = 100 µm. Statistical analyses were performed using one-way ANOVA with Tukey's multiple-comparison test (a-f). Error bars represent mean ± SEM.

bioRxiv preprint doi: https://doi.org/10.64898/2026.04.10.716420; this version posted April 14, 2026. The copyright holder for this preprint (which was not certified by peer review) is the author/funder, who has granted bioRxiv a license to display the preprint in perpetuity. It is made available under aCC-BY 4.0 International license.

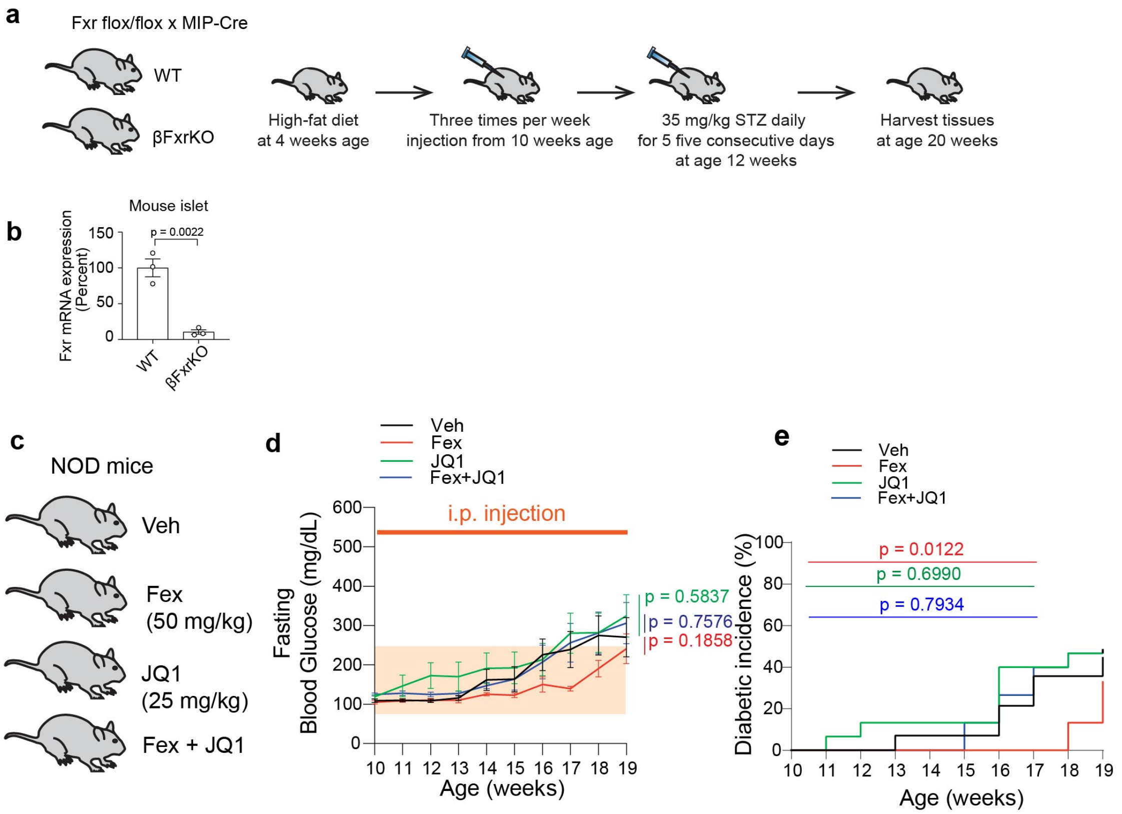

Supplementary Fig. 9. FXR agonist slow-down the progression of spontaneous T1D in NOD mice.
a, Schematic of the T2D model. C57BL/6J or βFxrKO mice were fed a HFD starting at 4 weeks of age. Pre-treatment was initiated at 10 weeks, followed by MLD-STZ administration for five consecutive days starting at 12 weeks. **b**, qPCR analysis of Fxr target gene expression in isolated islets from WT and βFxrKO mice. **c**, Scheme of mouse experiments. Veh, Fex (50 mg/kg), JQ1 (25 mg/kg) or Fex (50 mg/kg) + JQ1 (25 mg/kg) are i.p. injected daily from 10 weeks to 19 weeks old NOD female mice. **d**, Fed *ad lib* blood glucose levels (mg/dL). n = 5. **e**, Diabetic incidents (% > 250 mg/dL blood glucose levels). Statistical analyses were performed using unpaired two-tailed Student's t-test for (b), two-way ANOVA for (d), and Kaplan–Meier analysis with the log-rank (Mantel–Cox) test for (e). Error bars represent ± SEM.

bioRxiv preprint doi: https://doi.org/10.64898/2026.04.10.716420; this version posted April 14, 2026. The copyright holder for this preprint (which was not certified by peer review) is the author/funder, who has granted bioRxiv a license to display the preprint in perpetuity. It is made available under aCC-BY 4.0 International license.

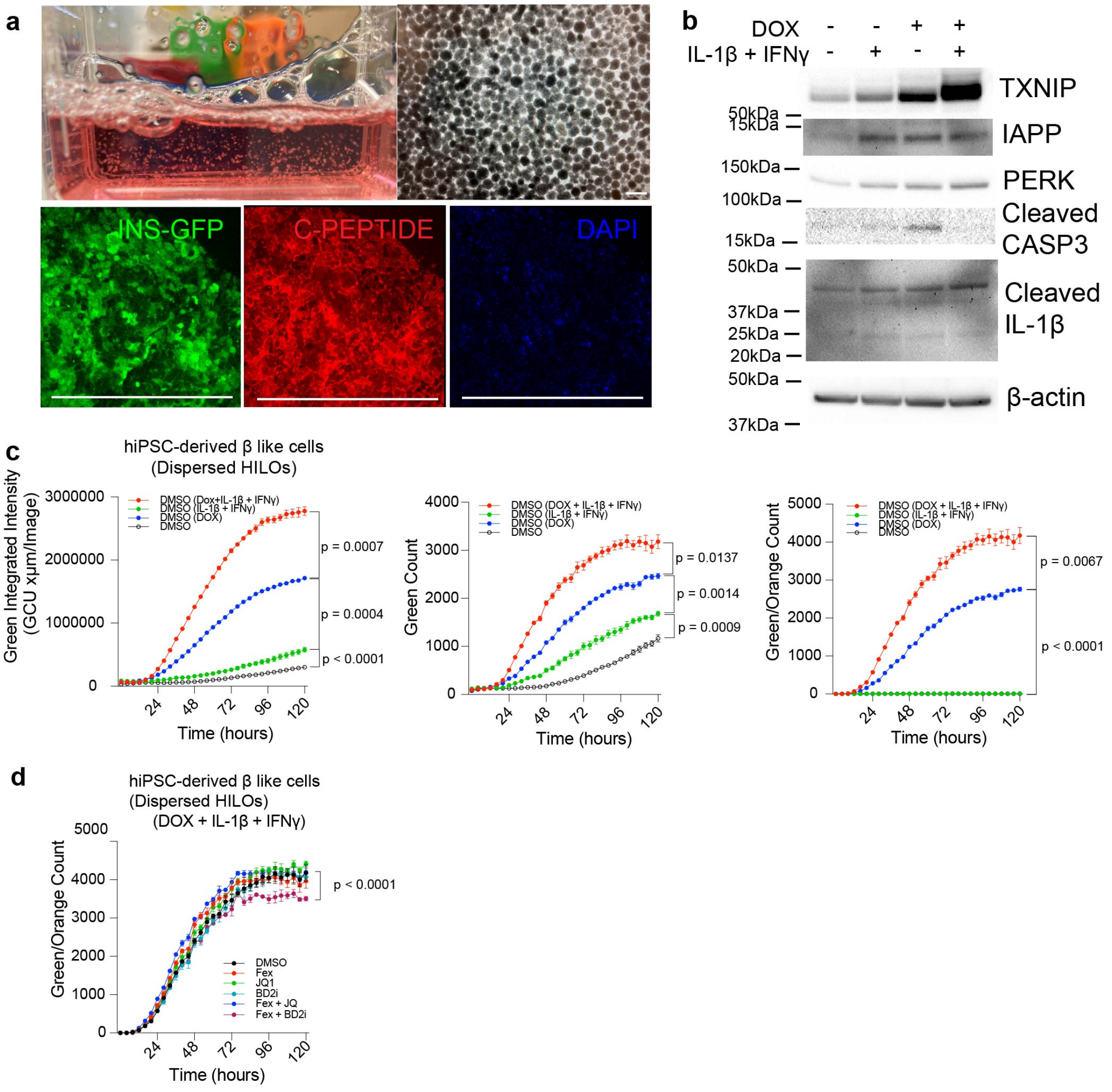

Supplementary Fig. 10. Model of human T2D in HILOs.

a, Representative image of 3D culture system to generate HILOs. Scale bar = 50 μm. **b**, DOX-inducible human TXNIP and human IAPP overexpression in dispersed HILOs. IB for TXNIP, Amylin (IAPP), PERK, cleaved caspase 3 and β-actin. **c**, Apoptosis rate in dispersed HILOs was measured by real-time imaging using Incucyte SX5. Dispersed HILOs were treated with DOX (1 μg/μl), IL-1β (10 ng/ml), and IFNγ (10 ng/ml) for 120 hours. n = 3/each. Apoptosis rate was determined by green-integrated intensity, green count and orange-green count (DOX-inducible TXNIP/IAPP coexpress mCherry and it's overlapping to Caspase 3/7 green fluorescence) respectively. n = 3/each. **d**, Apoptosis rate in dispersed HILOs was measured by real-time imaging using Incucyte SX5. Dispersed HILOs were treated with Fex (10 μM), JQ1 (500 nM), or iBET-BD2 (1 μM) under the stimulation of DOX (1 μg/μl), IL-1β (10 ng/ml), and IFNγ (10 ng/ml) for 120 hours. n = 3/each. Apoptosis rate was determined by orange-green count (DOX-inducible TXNIP/IAPP coexpress mCherry, which is colocalized with Caspase 3/7 green fluorescence) respectively. n = 3/each. Statistical analyses were performed using one-way ANOVA with Tukey's multiple-comparison test for (c, d). Error bars represent mean ± SEM.
